# Nosocomial Myiasis in an Intensive Care Unit (ICU): A Case Report

**Published:** 2019-06

**Authors:** Ehsan AHMADPOUR, Mohammad Reza YOUSSEFI, Mohammad NAZARI, Seyed Abdollah HOSSEINI, Arash RAKHSHANPOUR, Mohammad Taghi RAHIMI

**Affiliations:** 1. Infectious and Tropical Diseases Research Center, Tabriz University of Medical Sciences, Tabriz, Iran; 2. Department of Veterinary Parasitology, Babol Branch, Islamic Azad University, Babol, Iran; 3. Tuberculosis Laboratory, Deputy of Public Health of Mazandaran Province, Sari, Iran; 4. Student Research Committee, Toxoplasmosis Research Center, Mazandaran University of Medical Sciences, Sari, Iran; 5. Department of Medical Parasitology and Mycology, School of Public Health, Tehran University of Medical Sciences, Tehran, Iran; 6. School of Medicine, Shahroud University of Medical Sciences, Shahroud, Iran

**Keywords:** Nosocomial myiasis, Infestation, *Lucilia sericata*, Hygiene, Iran

## Abstract

Myiasis is a parasitic infestation of human and animal tissues caused by larva of several fly species. Nosocomial myiasis is a type of myiasis that patient acquires infestation after admission to hospital and prevention of this infestation should be one of hospital authorities concerns. Therefore, we report a case of nosocomial oral myiasis caused by *Lucilia sericata* in a woman aged 78-yr-old hospitalized in a Heart Center in north of Iran Aug 2015. The significance of fly extermination in hospital was highlighted and emphasized. Besides, the etiology and different aspects of infestation were discussed elaborately.

## Introduction

Myiasis, derived from the Greek (myia= mucosa) firstly was proposed by Hope in 1840 to define infestation of humans and animals by larva of dipterous. A wide range of animal species including human can be affected by myiasis. This infestation can cause multiple symptoms depending on the body location, species and number of larvae. Human myiasis is distributed throughout the world, particularly in poor socioeconomic regions and low hygiene conditions (1, 2).

The anatomical and ecological classifications are two main systems used to categorize myiasis. Myiasis of the oral cavity is a type of wounds caused by number of fly species in the order Diptera considered as an accidental occurrence and associated with poor oral hygiene, senility, gingival disease, trauma, and mental debility. Oral myiasis occurs due to direct infestation. It may be transmitted via contaminated food. This disease with varying clinical features, affect the lips and tongue (1, 3). Diagnosis is based on the history, clinical findings, observation and identification of larva. In addition, painful and enlarged mouth, teeth, gingiva, and feeling of movement, are probably symptoms described by oral myiasis. If oral myiasis diagnosed in the early stage can be absolutely benign and asymptomatic, but delay in diagnosis causes a serious problem because of the larvae penetration into the tissues. This is particularly important to at-risk individuals. Hospital-acquired myiasis (nosocomial infection) is rarely reported, being acquired in nursing homes especially in immobile and debilitated patients (4). Nosocomial myiasis has been reported in the United States, Europe and Iran (5–8). Nevertheless, records are incomplete and presumably, a number of cases as lost and not reported.

In this report, we describe a case of nosocomial which is the first case of nosocomial oral myiasis caused by *Lucilia sericata* in a woman from north of Iran.

## Case report

A female aged 78-year-old who resided in Mazandaran Province, Iran was admitted with cardiac arrest to a Coronary Care Unit (CCU) of Heart Centers in north of Iran, Aug 2015. Due to worse state of consciousness and clinical conditions, the patient was transferred to Intensive Care Unit (ICU) and intubated.

**Fig. 1:**
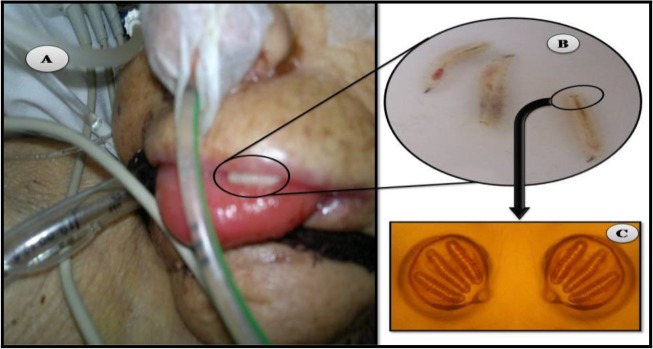
Patient’s with nosocomial oral myiasis: (A) Presence of larvae between upper lip and tongue. (B) The collected larvae of L. sericata from patient’s oral cavity. (C) The posterior spiracle of third instars of *L. sericata*

On the fifth day after admission, on examination of buccal cavity, some tiny worm-like creature moving around inside were observed on tongue and upper lip of the patient; eight wormlike bodies were collected from buccal cavity of the patient by a forceps and transferred to Department of Parasitology, Babol Branch Islamic Azad University for examination and identification (Fig. 1, A). Besides, no lesion and bleeding were observed on examination of patient buccal cavity. Surprisingly enough, by wandering the environment a fly was observed while roaming freely in the ICU. Although all of windows were equipped with net in order to exclude insects some of them were torn partly.

The white and motile collected bodies which ranged from 8 to12 mm identified as third instar transition of *L. sericata* larvae (green bottle fly) based on the morphological examination of posterior spiracles of larvae using Walker identification key (8) (Fig. 1, B and C). On admission, except the cardiac arrest, no abnormality was found in physical examination and all results of routine laboratory examinations were normal. In addition, the data obtained from the patient history indicated that she did not suffer any underlying diseases such as diabetes.

Informed consent was taken from the patient before the study and the study was approved by local Ethics Committee (IR.SHMU.REC.1397.040).

## Discussion

Myiasis is a more common disease in tropical and subtropical regions of the world. Animals are the most regular victims of myiasis but it can also affect human beings. Magen presented the first report of myiasis caused by *L. sericata* from eyes, mouth and paranasal sinuses of a hospital patient. This fly belongs to Calliphoridae family and they consider as ectoparasites in both humans and animals. Moreover, it is responsible for the most frequent causal agent of tissue, cavity, ocular and urogenital human myiasis in Central Europe. So far, several cases of human myiasis were reported but nosocomial myiasis is rare and unique even in endemic areas (1, 4). Minar and Vakoun were the first researchers who reported nosocomial myiasis caused by *L. sericata* in Iran (7). Iran is situated in sub-tropical area and numerous cases of nosocomial myiasis have been reported in different regions of Iran (4, 9, 10). Multiple cases of myiasis in Iran were identified and categorized clinically as furuncular, wound, ophthalmic, auricular, nasopharyngeal, oral, intestinal and genitourinary. Etiological agents of myiasis occurred in Iran are belonging to Calliphoridae, Sarcophagidae, Oestridae and Syrphidae flies (4).

Nosocomial myiasis is facultative and accidental infestation of nonliving organic matter and infrequently living tissue, such as necrotic tissues, open neglected wounds, ulcerating lesions and body cavity (nose, eye, and rarely the oral cavity) (11, 12). Like the other predisposing factors (mental retardation and hemiplegia), patients in comatose state is susceptible to suffering the oral myiasis like the present case (1, 4, 5). Nosocomial myiasis caused by facultative agents can be fatal and life treating (13). Hence, it is necessary to take care of the patients admitted to clinics in hope of getting rid of their problems but sometimes they acquire new infection agents such as nosocomial myiasis. Therefore, hospital rooms must be kept free of flies. Larvae can enter through tissues; so early diagnosis of myiasis seems to preclude the larvae penetration and consequently extensive tissue damage and morbidity.

In the current case, on physical examination of buccal cavity of the patient no lesion and bleeding were observed (1–4). Observation of larva from buccal cavity of the patient occurred on the fifth day after admission to the clinic and based on the life cycle of *Lucilia* fly the requisite time for egg hatching to conical larva and completing peritreme of posterior respiratory spiracles is almost 8–12 h and after 4–12 d larva develop and transform into adult flies, it seems reasonable to infer that oral myiasis of the patient happened in the hospital when she was hospitalized. In addition, observation of the fly was another document, which confirms this significance. Therefore, this myiasis is considered as nosocomial myiasis.

## Conclusion

Nosocomial myiasis usually occurs in susceptible groups including immobile, severely ill, semi-conscious and comatose patients and these individuals are more at risk to nosocomial myiasis. Prevention of nosocomial myiasis is possible by installing screens with tiny mesh size in front of hospital windows to exclude flies and extermination of the flies by insecticides.

## Ethical considerations

Ethical issues (Including plagiarism, informed consent, misconduct, data fabrication and/or falsification, double publication and/or submission, redundancy, etc.) have been completely observed by the authors.

## References

[1] Francesconi F Lupi O (2012). Myiasis. Clin Microbiol Rev, 25:79–105. 22232372 10.1128/CMR.00010-11 PMC3255963

[2] Babamahmoudi F Rafinejhad J Enayati A (2012). Nasal myiasis due to Lucilia sericata (Meigen, 1826) from Iran: a case report. Trop Biomed, 29(1):175–9. 22543618

[3] Tirgari S Nateghpour M Jahanian AH Akbarzadeh K (2003). Case report: First Record of Human Myiasis caused by *Chrysomia bezziana* (Villeneuve) in Iran (Diptera, Calliphoridae). Iran J Public Health, 32(3), 68–70.

[4] Alizadeh M Mowlavi G Kargar F (2014). A Review of Myiasis in Iran and a new Nosocomial Case from Tehran, Iran. J Arthropod Borne Dis, 8(2):124–31. 26114125 PMC4478423

[5] Youssefi MR Rahimi MT Marhaba Z (2012). Occurrence of Nasal Nosocomial Myiasis by *Lucilia sericata* (Diptera: Calliphoridae) In North of Iran. Iran J Parasitol, 7: 104–8. PMC3488829 23133480

[6] Ahmadi MS Nasirian H Gheshmi AN Ershadi MY (2009). Human extensive head Skin myiasis. Iran J Public Health, 38(1): 134–38.

[7] Minar J Herold J Eliskova J (1995). Nosocomial myiasis in Central Europe. Epidemiologie, mikrobiologie, imunologie: casopis Spolecnosti pro epidemiologii a mikrobiologii Ceske lekarske spolecnosti. J E Purkyne, 44: 81–3. 7670806

[8] Leylabadlo HE Kafil HS Aghazadeh M Hazratian T (2015). Nosocomial oral myiasis in ICU patients: occurrence of three sequential cases. GMS Hyg Infect Control, 10: Doc16. 26682129 10.3205/dgkh000259 PMC4672872

[9] Walker A (1994). The arthropods of human and domestic animals: a guide to preliminary identification. Springer Company; 112–15.

[10] Maleki Ravasan N Shayeghi M Najibi B Oshaghi MA (2012). Infantile Nosocomial Myiasis in Iran. J Arthropod Borne Dis, 6: 156–63. 23378974 PMC3547307

[11] Dutto M Pellegrino M Vanin S (2013). Nosocomial myiasis in a patient with diabetes. J Hosp Infect, 83:74–76. 23149058 10.1016/j.jhin.2012.08.019

[12] Najjari M Shafiei R Fakoorziba MR (2014). Nosocomial myiasis with Lucilia sericata (Diptera: Calliphoridae) in an ICU patient in Mashhad, Northeastern of Iran. Arch Iran Med, 17(7):523–5. 24979568

[13] Mowlavi G Nateghpour M Teimoori S (2011). Fatal nosocomial myiasis caused by *Lucilia sericata*. J Hosp Infect, 78(4):338–9. 21684632 10.1016/j.jhin.2011.04.005

